# Survival, stent patency, and cost‐effectiveness of plastic biliary stent *versus* metal biliary stent for palliation in malignant biliary obstruction in a developing country tertiary hospital

**DOI:** 10.1002/jgh3.12618

**Published:** 2021-07-20

**Authors:** Muthia Farani, Siti R F Saldi, Hasan Maulahela, Murdani Abdullah, Ari F Syam, Dadang Makmum

**Affiliations:** ^1^ Department of Internal Medicine Faculty of Medicine Universitas Indonesia, Cipto Mangunkusumo General Hospital Jakarta Indonesia; ^2^ Clinical Epidemiology and Evidence‐Based Medicine Unit Faculty of Medicine Universitas Indonesia, Cipto Mangunkusumo General Hospital Jakarta Indonesia; ^3^ Division of Gastroenterology, Department of Internal Medicine Faculty of Medicine Universitas Indonesia, Cipto Mangunkusumo General Hospital Jakarta Indonesia

**Keywords:** biliary stent, cost‐effectiveness, malignant biliary obstruction

## Abstract

**Background and Aim:**

Patients with advanced malignant obstructive jaundice often require biliary drainage. Resources restraint makes clinicians need to outweigh effectiveness of each biliary stents and their costs. Hence, a cost‐effectiveness analysis is necessary.

**Methods:**

A retrospective cohort study was done on malignant biliary obstruction patients undergoing palliative biliary stenting between January 2015 and December 2018. We evaluated 180‐day survival rate using log‐rank test and stent patency duration using Mann–Whitney *U* test. Effectiveness was defined as stent patency, while cost was calculated using hospital perspective using decision tree model and reported as incremental cost‐effectiveness ratio.

**Results:**

A total of 81 men and 83 women were enrolled in this study. One hundred and eighty days survival rate was 35.9% (median 76 days, 95% confidence interval [CI] 50–102 days) and 33.3% (median 55 days, 95% CI 32–78 days), while average stent patency was 123 (8) days *versus* 149 (13) days for plastic and metal stent groups, respectively (*P* > 0.05). Metal stent could save Indonesian Rupiah (IDR) 1 217 750 to get additional 26 days of patency.

**Conclusion:**

There were no differences in survival and stent patency between the two groups. Metal biliary stent is more cost‐effective than plastic stent for palliation in malignant biliary obstruction.

## Graphical abstract

This study describes the cost‐effectiveness, stent patency, and survival comparison between plastic stent and metal stent for palliation of pancreatobiliary malignancy in a developing country.

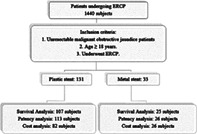

## Introduction

Obstructive jaundice is often encountered in patients with pancreatobiliary malignancy. Its adverse effects on patients include pruritus, malabsorption, coagulopathy, as well as more severe complications such as cholangitis and biliary sepsis, which can further lead to multi‐organ dysfunction, even death.^1^ The main modality for management of malignant obstructive jaundice due to pancreatobiliary malignancy is tumor resection, but in case of unresectable tumor, the treatment given to patient is palliative. One of the palliative treatments is biliary drainage to prevent complications of obstructive jaundice. Biliary drainage can be achieved by inserting biliary stent, either made by metal or plastic, through endoscopic retrograde cholangiopancreatography (ERCP) procedures, percutaneous transhepatic biliary drainage (PTBD), and biliary bypass surgery.^2^, ^3^, ^4^

The life expectancy of pancreatobiliary malignancies is abysmal, largely because 85% of patients seeking for help are already at advanced stage, making curative therapy impossible.^5^ The median survival of patients after tumor resection was 25–34 months, while the median survival in unresectable cases was only 4.7 months.^6^, ^7^ Patients who underwent per‐endoscopic stent insertion had a median of 81‐day survival.^8^

In the era of universal health coverage (also called Jaminan Kesehatan Nasional [JKN] in Indonesia), case‐based groups tariff is applied as the payment method. Any discrepancies between the coverage and the actual expense are subject to healthcare providers.^9^ Therefore, healthcare providers are required to be efficient in every aspect of health service while also maintaining patient safety. Palliative management in patients with malignant obstructive jaundice also need to consider the cost‐effectiveness of each given treatments due to patient's short survival. A cost‐effectiveness analysis is needed to investigate which treatment option resulting in the highest health effect with the least cost.

Studies have demonstrated cost‐effectiveness of metal stent compared with plastic stent for palliation in malignant biliary obstruction due to its longer patency rate,^10^, ^11^ although there is an inconsistence between studies.^12^ There are no data in Indonesia regarding the survival and duration of stent patency in association with the cost‐effectiveness of plastic stent compared with metal stent insertion as palliative management in patients with malignant obstructive jaundice. Thus, the aim of this study is to compare the survival rate, stent patency, and cost‐effectiveness of plastic and metal stents as palliative management for patients with malignant obstructive jaundice.

## Methods

We conducted a retrospective cohort of malignant obstructive jaundice patients who underwent biliary stent insertion through ERCP procedures as a palliative treatment at Gastrointestinal Endoscopic Center (GEC/PESC) Cipto Mangunkusumo General Hospital, Indonesia, between 1 January 2015, and 31 December 2018. The inclusion criteria of this study were unresectable malignant biliary obstruction, age ≥ 18, and patients underwent biliary insertion through ERCP. The exclusion criteria were patients with insufficient data. The sampling method for this study was total sampling with a minimum of 64 subjects in each group, using secondary data obtained from electronic and conventional medical record. The subjects of this study were observed for 6 months after a stent insertion.

Survival and patency outcome were presented in the Kaplan–Meier curve. Subjects who were lost to follow‐up and did not experience the event at the end of the observation were counted as censored. The difference of survival and patency rates between the two groups was analyzed using a log‐rank test. The difference of stent patency duration was analyzed using Mann–Whitney *U* test. Overall cost was calculated using hospital perspective (direct medical costs including medication, imaging, laboratory, surgery and other medical interventions, inpatient and outpatient costs) and following a decision tree model. Cost‐effectiveness was presented as incremental cost‐effectiveness ratio (ICER).

This study was approved by ethical health research committee of Faculty of Medicine, Universitas Indonesia, Cipto Mangunkusumo National General Hospital with letter number 686/UN2.F1/ETIK/PPM.00.02/2019. This study was solely observational, and thus would not compromise the treatment of patients.

## Results

**Figure 1:**
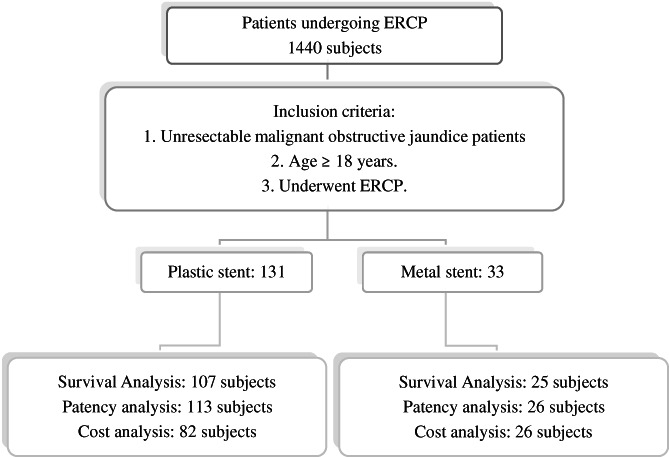
Subjects enrollment graph. ERCP, endoscopic retrograde cholangiopancreatography.

There were 1,440 patients undergoing biliary stent insertion through ERCP in GEC, Cipto Mangunkusumo General Hospital during this study period. Among those patients, 268 patients met the inclusion criteria. A total of 104 patients had missing data in their medical record, therefore 164 subjects were included in this study. Subject recruitment process can be found in Figure 1.

A total of 81 men and 83 women aged 24–88 years of age were enrolled in this study. The most common etiology was pancreatic head cancer (42.7%). There were no differences of characteristics except a small difference in liver metastasis and admission route proportion between two groups. Patients' characteristics of this study are shown in Table 1.

**Table 1:** Patient characteristics

Characteristics	Plastic stent (*n* = 131)	Metal stent (*n* = 33)	*P* value
Age, *n* (%)			0.614
<60 years old	69 (52.7)	19 (57.6)	
≥60 years old	62 (47.3)	14 (42.4)	
Sex, *n* (%)			0.199
Male	68 (51.9)	13 (39.4)	
Female	63 (48.1)	20 (60.6)	
Diagnosis, *n* (%)			0.189
Malignant neoplasm of head pancreas	54 (41.2)	16 (48.5)	
Malignant neoplasm of ampulla vater	12 (9.2)	2 (6.1)	
Cholangiocarcinoma	45 (34.4)	11 (33.3)	
Malignant neoplasm of gall bladder	1 (0.8)	2 (6.1)	
Other	19 (14.5)	2 (6.1)	
Comorbidity, *n* (%)			0.584
CCI < 4	47 (35.9)	10 (30.3)	
CCI ≥ 4	84 (64.1)	23 (69.7)	
Liver metastasis, *n* (%)	63 (48.1)	18 (54.5)	
Admission, *n* (%)			0.312
Fast track	35 (26.7)	6 (18.2)	
Emergency room	96 (73.3)	27 (81.8)	
Cholangitis, *n* (%)			0.848
Yes	50 (38.2)	12 (36.4)	
No	81 (61.8)	21 (63.6)	
Number of interventions in 6 months, *n* (%)			0.002
Once	86 (65.6)	32 (97)	
Twice	41 (31.3)	1 (3)	
>Twice	4 (3.1)	0 (0)	
Initial total bilirubin level (g/dL), median (IQR)	20.16 (15.74)	23.16 (20.69)	0.680
Initial albumin level, *n* (%)			0.281
<3.5 g/dL	112 (85.5)	25 (75.8)	
≥3.5 g/dL	19 (14.5)	8 (24.2)	
Length of stay (days), median (IQR)	14 (17)	13 (14)	0.283
Survival observation range (days), mean (SD)	76.7 (64.9)	57.36 (62.0)	0.224
Stent patency observation range (days), mean (SD)	49.7 (48.2)	54.61 (63.0)	0.967

CCI, Charlson comorbidity index; IQR, interquartile range.

Mortality rate in the first month after the insertion in the plastic stent group was 25.2% and metal stent was 30.3%. Whereas in the 6‐month follow‐up, the mortality rates were 64.1 and 66.7% in the plastic stent and metal stent group, respectively. There were 32 lost‐to‐follow‐up subjects, therefore we did additional analysis according to best/worst case scenario to avoid under/overestimate the result. Cumulative survival proportion during 6 months of observation can be seen in Table 2.

**Table 2:** Cumulative survival proportion based on 180‐day observation

Survival day	Cumulative survival proportion (%)			
	Plastic stent *n* = 131		Metal stent *n* = 33	
	Best prediction	Worst prediction	Best prediction	Worst prediction
30	74.8	64.1	69.7	48.9
60	61.1	47.3	48.5	27.3
90	51.9	37.4	42.4	21.2
180	35.9	17.6	33.3	12.1

**Figure 2:**
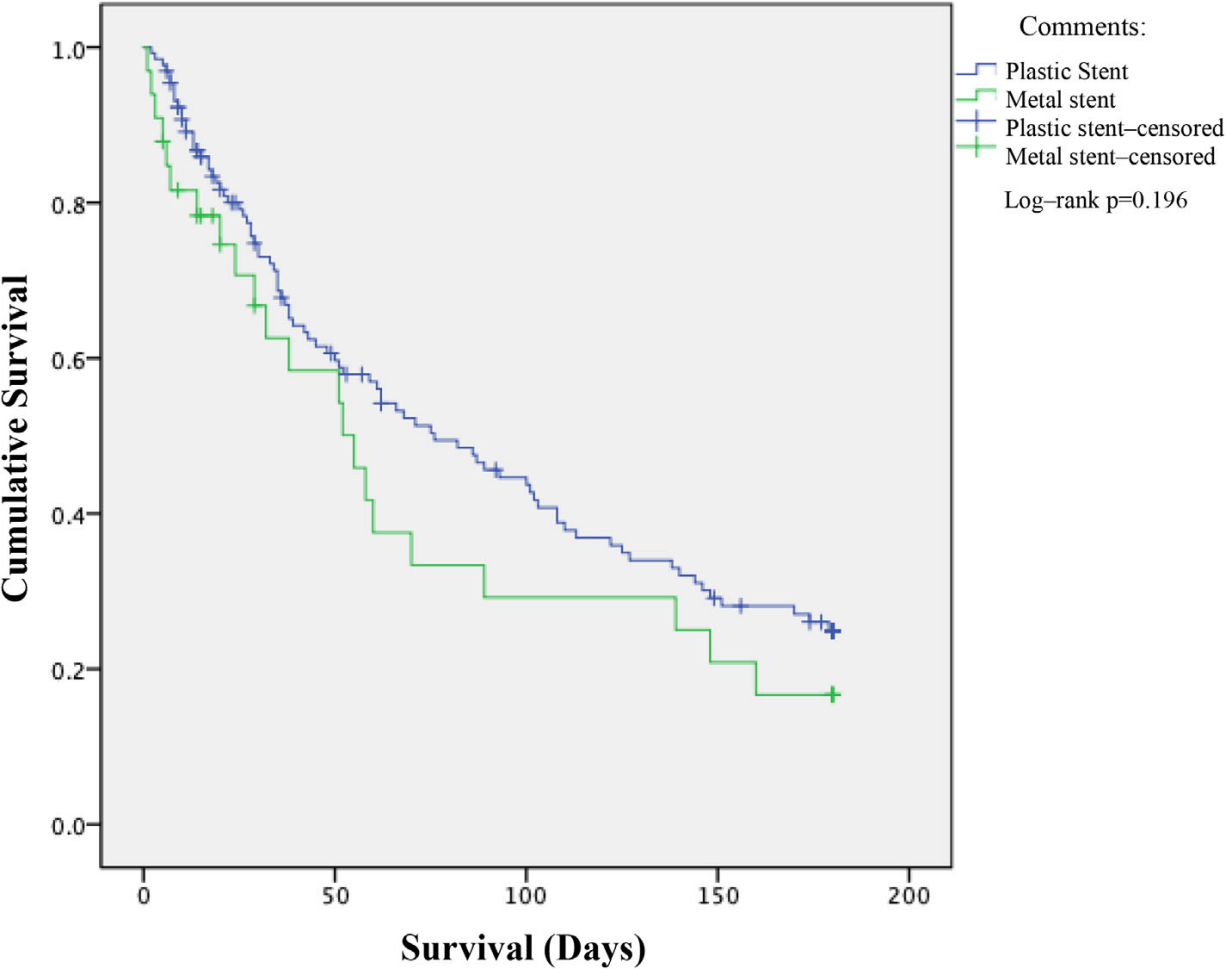
Survival function. Comments: (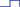), Plastic stent; (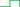), metal stent; (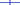), plastic stent‐censored; (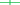), metal stent‐censored. Log‐rank *P* = 0.196.

Using Kaplan–Meier method, it was found that the median survival in the plastic stent group was 76 days (95% confidence interval [CI] 50–102 days), whereas in the metal stent group was 55 days (95% CI 32–78 days) (Fig. 2). In the log‐rank test, there was no significant difference in the survival of the two groups (*P* = 0.196). Overall median survival was 68 days (95% CI 44–92 days).

Additional analyses using Cox regression with backward method found that factors affecting survival were higher initial bilirubin level (hazard raio [HR] = 1.023; 95% CI 1.006–1.040; *P* = 0.006), initial albumin level < 3.5 (HR = 2.635; 95% CI 1.443–4.811; *P* = 0.002), fast track admission route (HR = 0.536; 95% CI 0.321–0.896; *P* = 0.017), and absence of liver metastasis (HR 0.590; 95% CI 0.397–0.877; *P* = 0.009). Adjusted hazard ratio for plastic stent to survival was 0.716 (95% CI 0.446–1.149; *P* = 0.166).

The 30‐day dysfunction rate after stent insertion was 13% in the plastic stent group and 12.1% in the metal stent group. The 60‐day and 90‐day dysfunction rates in plastic stent group were 16.8 and 19.1, respectively. There was no change in stent dysfunction rate at 60 and 90 days of follow‐up compared with 30‐day follow‐up. At the end of the observation, which was the 180th day, there were 24.4% plastic stents and 15.2% metal stents that were malfunctioning. Besides the lost‐to‐follow‐up subjects, patency outcome in plastic stent group was also affected by elective re‐intervention before the stent was malfunctioning. We did separate analysis to see stent patency proportion in full observation of 180‐day follow‐up (Table 3).

**Table 3:** Cumulative patent stent proportion based on 180‐day observation

Survival day	Cumulative patent stent proportion (%)			
	Plastic stent		Metal stent	
	All subjects *n* = 131	Fully observed subjects *n* = 80	All subjects *n* = 33	Fully observed subjects *n* = 80
30	87.0	78.8	87.9	84.6
60	83.2	72.5	87.9	84.6
90	80.9	68.8	87.9	84.6
180	75.6	60.0	84.8	80.8

**Figure 3:**
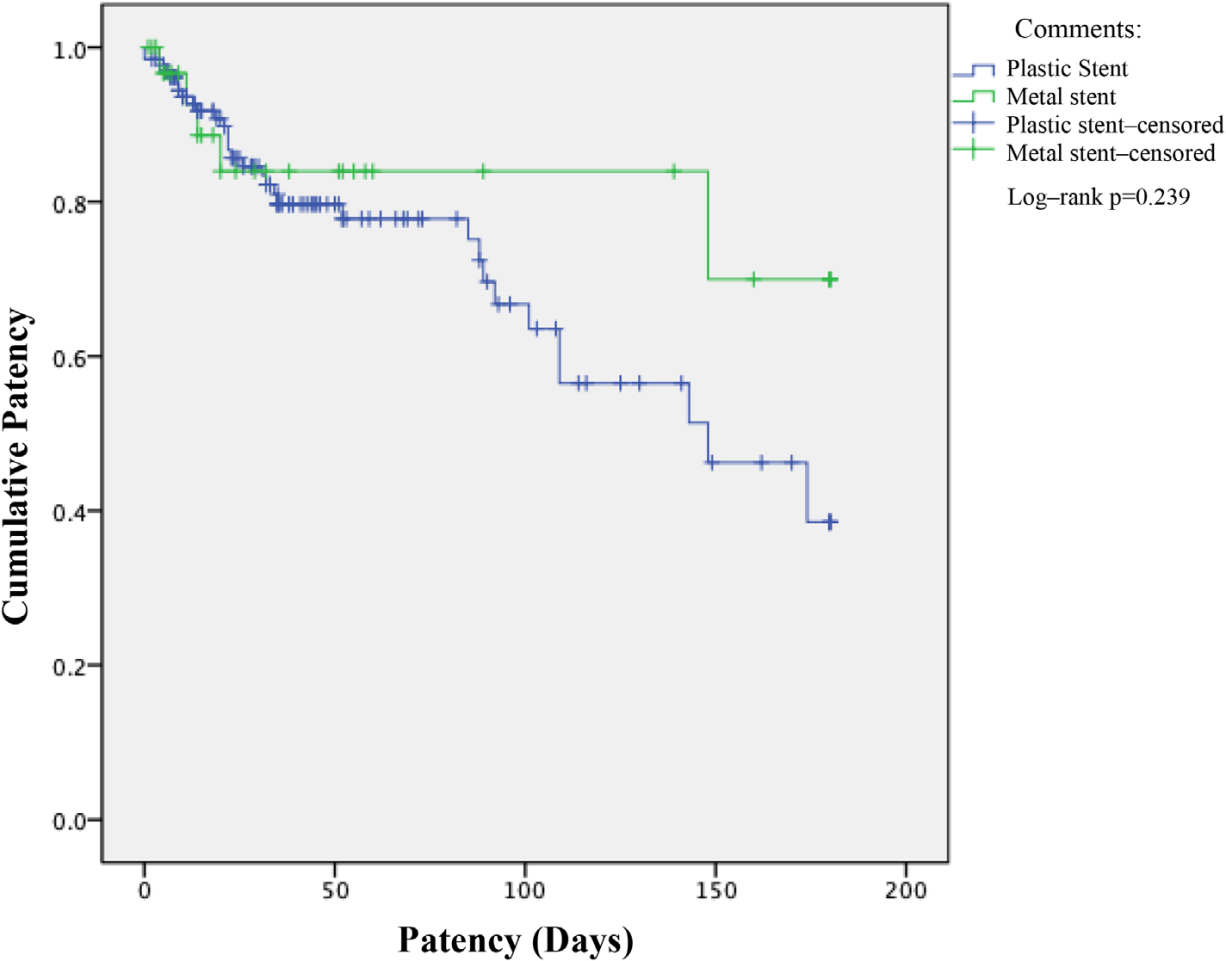
Patency function. Comments: (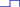), Plastic stent; (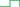), metal stent; (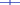), plastic stent‐censored; (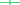), metal stent‐censored. Log‐rank *P* = 0.239.

Using the Kaplan–Meier method, the average patency of plastic stents was 123 days (95% CI ± 8 days), and the average patency for metal stents was 149 days (95% CI ± 13 days). There were no significant differences between the two groups (log‐rank test *P* = 0.239) (Fig. 3).

In the nonparametric test using the Mann–Whitney *U* test, the duration of stent patency of the two groups also did not reveal any significant difference (*P* = 0.489).

**Figure 4:**
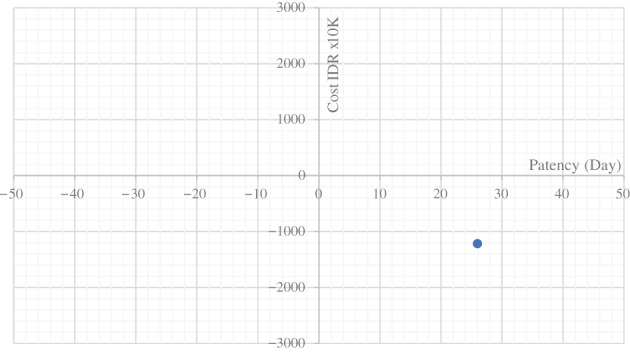
ICER cost–patency.

We excluded lost‐to‐follow‐up subjects for cost analysis. Median (interquartile range [IQR]) of initial cost was IDR 60 663 260 (35 158 874) in the plastic stent group, and IDR 93 998 886 (54 128 899) in the metal stent group. Using a decision tree model, the average total cost in 6 months with stent patent outcome for the two groups were respectively IDR 105 571 028 (proportion of patent stent was 67%) and IDR 104 446 233 (proportion of patent stent was 81%). The incremental costs for inserting metal stent was IDR 1 217 750 for 26 days additional patency. The ICER values were plot in the coordinates and fell to the right lower quadrant. This showed that the use of metal stent was dominant than the use of plastic stent (Fig. 4).

## Discussion

Endoscopic biliary drainage is one of palliative treatments for pancreatobiliary malignancy. The choice between plastic stent and self‐expandable metallic stent (SEMS) depends on patient prognosis and cost (especially in developing countries). Patient with longer survival preferably using SEMS compare to plastic stent. The survival rate at the end of observation (180‐day) in this study was 35.9% in the plastic stent group and 33.3% in the metal stent group. Median survival in this study was lower than in the meta‐analysis conducted by Almadi *et al*. in 2017, involving 20 randomized controlled trials with a total of 1713 subjects (114–267 days *vs* 55 days and 48–228 days *vs* 76 days) in the metal and plastic stent groups, respectively.^13^

The difference in survival may be due to differences in the additional palliative management received by study subjects other than biliary stent insertion. Chemotherapy^14^ or chemoradiation^15^ was related to an increase in survival by 6.77–11.7 months and 12.9 months respectively. In accordance with these data, in this study, there were five subjects who received chemotherapy, with four of them living within 6 months. However, the number of subjects who received the chemotherapy was too small to be analyzed. Although gemcitabine is the drug of choice during chemotherapy of patients with pancreatobiliary malignancies,^2^, ^3^, ^4^ the number of subjects in this study who received chemotherapy is very small possibly due to Government's restrictions on the use of gemcitabine in pancreatobiliary malignancies.^16^ Based on the Decree of the Minister of Health of Republic of Indonesia number 01.07 of 2018 about national drug formulary, gemcitabine can only be used for unresectable pancreatic adenocarcinoma patients who have received 5‐FU. Chemotherapy indicates good performance status. With regard to survival, patients with better performance status have a longer survival. Wilcox *et al*. in his study conducted in America found a significant difference in the median survival between patients with plastic and metal stents, 2.8 months compared with 11.6 months (*P* < 0.0001). In that study, patients with Karnofsky score < 80 and/or liver metastases were given intervention of plastic stent insertion, while patients with Karnofsky score ≥ 80 were given intervention of metal stent insertion. In subgroup analysis, the median survival rate of patients with a Karnofsky score < 80 was 93 days.^17^ However, because this study was retrospective, Karnofsky score or other performance status could not be presented. Another study by Katsinelos *et al*. also found that the survival of malignant obstructive jaundice patients with liver metastasis was shorter than without liver metastasis (52 days compared with 329 days).^18^ Such results are not much different from the median survival in this study, which was 68 days (95% CI 44–92 days), in which nearly half of the subjects (49.4%) had liver metastases. This is also supported by the results of the Cox regression test in this study, which obtained liver metastasis as a predictor of poor survival (HR 0.651; 95% CI 0.433–0.979; *P* = 0.39). The proportion of patients with liver metastasis in this study was more in the metal stent than in plastic stent group (54.5 *vs* 48.1% *P* = 0.507). This might cause the patient survival in the metal stent group to be shorter than that of the plastic stent group in this study.

Based on patients' admission route, we grouped patients into *fast track* groups, namely the path of care to speed up patients who would undergo medical procedures with relatively good condition, as well as the emergency group in patients with adverse conditions. In the metal stent group, the proportion of patients treated in the emergency room was more (81.8%) compared with the plastic stent group (73.3%). Of that number, 77.8 and 84.3%, respectively, in the metal and plastic stent groups experienced cholangitis. This indicates that patients in the plastic stent group had a relatively better clinical condition than the metal stent group.

In addition, there are several other factors that have been reported to affect the survival of unresectable malignant obstructive jaundice among other bilirubin levels before treatment^19^ and albumin levels.^8^, ^14^ Additional analyses found factors that influenced the survival in this study were initial bilirubin levels, initial albumin levels <3.5 g/dL, and fast track admission route.

The mean (SD) patency of the plastic stent group is 123 (8) days, and for the metal stent group it is 149 (13) days. In the nonparametric test using the Mann–Whitney *U* test, there was no significant difference in the duration of patency of the two groups (*P* = 0.489). This is consistent with previous studies' reports that metal stents have a better patency duration than plastic stents. Median patency of plastic and metal stents in the literature ranged between 35–165 days and 80–273 days, respectively.^11^, ^18^, ^20^, ^21^ In this study, the median plastic patency was 148 days (95% CI 84–212 days) while the median patency of metal stent was not achieved. The mean stent patency of the two groups in previous studies was 153–120 days for plastic stents and 273–385 days for metal stents.^11^, ^22^, ^23^ This difference with our result might be due to patient's short survival, hence the actual stent patency, especially for the metal stent, could not be observed properly. In reporting patency outcomes, there are two studies that distinguish the term “stent patency” from “stent failure.” Patency was only measured in subjects who were still alive during the observation, while stent failure was measured in both subjects who were still alive or had died during measurement. By means of the second measurement, Lammer *et al*. in their randomized controlled study in Austria obtained a median time to stent failure was 81 days and 122 days, compared with the stent patency results of 96 days and 272 days for the plastic and metal stent groups, respectively.^20^, ^30^ Similar results were also obtained for a study performed in Japan for pancreatic head cancer patients, where the median time to stent malfunction is 110 (0–425) days for the plastic stent group, and 144 days (2–415 days) for the stent metal group. Shorter results were obtained when compared with patency outcomes in living subjects in both groups in that study with a median patency of 133 (1–429) days for plastic stent and 419 (2–536) days for metal stent.^23^ In this study, patency was measured by seeing recurrent jaundice, both when the patient is still alive during observation and when the patient dies.

The occlusion mechanism in plastic biliary stent is different from metal biliary stent. In plastic stent, the most widely accepted theory is the formation of bacterial biofilms and sludges filling the stent lumen, both with and without infection, and duodeno‐biliary reflux that contains food fiber.^24^, ^25^, ^26^ Although more commonly found on plastic stents, the formation of bacterial and sludge biofilms and duodeno‐biliary reflux can also occur in metal stents.^23^ Larger stent diameter is associated with better patency because it takes longer time for biofilms or sludges to totally block the stent lumen.^24^, ^27^ However, in prospective and retrospective studies, there were no significant differences in the patency duration of plastic stents with a diameter of 7 Fr, 10 Fr, and 11 Fr.^28^, ^29^ In accordance with that result, we also found no difference in plastic stent patency by adjusting the stent diameter. The presence of side holes in the stent is also associated with higher biofilm and sludge growth.^24^, ^27^ A study by Katsinelos *et al*. in Japan obtained a median patency of Tannenbaum stent (plastic stent designed without side holes), which was 123.5 days.^18^

Another consideration is that the cause of stent malfunction is not only occlusion, but also migration of the stents. Migration of plastic stent is associated with stent anatomy. A pigtail will decrease the incidence of migration, but all plastic biliary stents with pigtails have side holes, which has been discussed previously to be associated with higher occlusion rates.^24^

Occlusion mechanism in metal stent is more often caused by tumor ingrowth, especially in uncovered types. Efforts to reduce this are done by making the covered or partially covered biliary stents. Despite having a low number of ingrowth tumors, covered and partially covered biliary metal stents are more likely to migrate due to tumor growth outside the stents (overgrowth).^27^

The number of interventions in the plastic stent group is greater than in the metal stent group. But this is not only due to differences in patency of the two stents that have been consistently reported in various studies.^11^, ^18^, ^20^, ^21^, ^22^, ^23^ In the plastic stent group, an elective re‐intervention was performed, aimed at preventing cholangitis due to stent occlusion. There were 15 (34%) of 44 patients in the plastic stent group undergoing elective re‐intervention. Whereas in the stent metal group, only one patient out of three people who experienced stent failure underwent re‐intervention.

The median (IQR) of the initial cost for plastic biliary stent group was IDR 60 663 260 (35 158 874), and for metal stent group it was IDR 93 998 886 (54 128 899). After 6 months of observation, the average cost of the plastic stent group with patent stent outcome was IDR 105 571 028 (proportion of 67%) and IDR 104 446 233 (proportion 81%) for metal stent group. Incremental cost‐effectiveness for biliary metal stent insertion was IDR 1 217 750 to get additional stent patency of 26 days. This is consistent with the results of several previous studies that found metal stents to be more cost‐effective than plastic stents.^10^, ^11^, ^12^, ^30^ However, two of those studies did not use the actual cost‐effectiveness analysis method.^10^, ^11^ Higher cost in the plastic stent group is not only caused by re‐intervention due to stent malfunction, but also the planned (elective) re‐interventions carried out as an effort to prevent cholangitis due to stent failure. Elective re‐intervention is carried out every 3 months. Given the median survival of patients with 76 plastic stents (50–102 days) with a mean patency of 123 (8 days), patients in the plastic stent group are likely to get re‐intervention at least once even though the stent condition is still patent.

Another thing to consider when applying the result of this cost‐effectiveness analysis, especially in a Government Hospital to daily basis, is the Indonesian Cased Based Groups or INA‐CBG as a standard tariff paid by BPJS to health services. As a national referral hospital, Cipto Mangunkusumo General Hospital has the highest INA‐CBG package rate than other hospitals. However, tariffs based on the INA‐CBG package follow a diagnosis and/or medical procedures input (coding) based on ICD 10. Mild, moderate, and severe hepatobiliary and pancreatic system tumors have a tariff of IDR 6 763 200, IDR 9 468 500, and IDR 11 470 900, respectively. While mild, moderate, and severe bile duct complex procedures have a tariff of IDR 32 192 200, IDR 38 858 700, and IDR 59 275 000.63, respectively. Unfortunately, ERCP procedures are not counted as “bile duct complex procedures” in INA‐CBG, so the cost of malignant obstructive jaundice patients undergoing ERCP only follows package rates based on the diagnosis code. Patients with pancreatobiliary malignancies with ERCP and stent placement without comorbid diseases and complications are included in the moderate hepatobiliary and pancreatic system tumor diagnosis, so the rate paid by Government is only IDR 9 468 500. With a plastic stent insertion rates of IDR 20 812 500 and metal stent IDR 42 092 000, the minimum discrepancy that will be subjected to the hospital is IDR 11 344 000 and IDR 32 523 500 for each type of stent, respectively. However, the impact of INA‐CBG rates on the health services loss requires a separate analysis.

Limitations of this study are the nature of retrospective research, which can lead to bias in patients' selection and information. Selection bias can be avoided by total sampling. Information bias because of non‐randomization subjects can be avoided by objective data to determine inclusion criteria in this study. This study was conducted in only one hospital, which is the national referral hospital in Indonesia, so the results obtained can be unrepresentative for similar patients in various other hospital types.

In conclusion, there were no statistically differences in survival and patency between plastic and metal biliary stents. Metal biliary stent is more cost‐effective than plastic stent for palliation in malignant biliary obstruction.
